# Uso da Ivabradina na Terapêutica de Pacientes Portadores da Síndrome da Taquicardia Postural Ortostática (POTS): Uma Revisão Sistemática

**DOI:** 10.36660/abc.20250347

**Published:** 2025-10-08

**Authors:** Ana Paula Giannella de Melo, Miguel Antônio Moretti, Antonio Carlos Palandri Chagas

**Affiliations:** 1 Faculdade de Medicina do ABC Santo André SP Brasil Faculdade de Medicina do ABC, Santo André, SP – Brasil; 2 Hospital das Clínicas Faculdade de Medicina Universidade de São Paulo São Paulo SP Brasil Hospital das Clínicas da Faculdade de Medicina da Universidade de São Paulo (HCFMUSP), São Paulo, SP – Brasil

**Keywords:** Ivabradina, Síndrome da Taquicardia Postural Ortostática

## Abstract

**Fundamento:**

A Síndrome da Taquicardia Postural Ortostática (POTS, do inglês *Postural Orthostatic Tachycardia Syndrome* ) é uma disfunção autonômica definida por sintomas de intolerância ortostática, associada à elevação da frequência cardíaca até 10 minutos após assumir a posição ortostática ou inclinação da cabeça para cima, na ausência de hipotensão. Existem três fenótipos de POTS (neuropática, hipovolêmica e hiperadrenérgica) e todos resultam em taquicardia e alteração da perfusão cerebral. A terapia farmacológica está indicada em alguns casos, mas ainda não foi aprovado um medicamento específico para essa condição. Alguns estudos mostraram que a ivabradina, pode ser benéfica, reduzindo a frequência cardíaca sem afetar a pressão arterial.

**Objetivo:**

Avaliar a eficácia e a segurança da ivabradina no tratamento da POTS.

**Método:**

Revisão sistemática com descritores “Ivabradina” e “Síndrome da Taquicardia Postural Ortostática” nas bases de dados PubMed, Scielo, LILACS e Google Scholar. Artigos agrupados e avaliados pela estratégia PICO.

**Resultados:**

Identificados 52 artigos, sete foram incluídos na revisão e separados em prospectivos^3^ e retrospectivos.^4^ No total, 203 pacientes foram avaliados, sendo a maioria do sexo feminino. Houve redução significativa da pressão arterial e melhora dos sintomas de intolerância ortostática em todos os estudos e a maioria dos pacientes não relatou efeitos adversos, independentemente do fenótipo de POTS do paciente.

**Conclusão:**

A ivabradina mostrou-se eficaz e segura na terapêutica dos pacientes portadores de POTS.

## Graphical abstract

**Figura Central:**
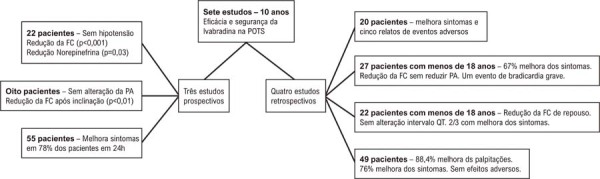
Uso da Ivabradina na Terapêutica de Pacientes Portadores da Síndrome da Taquicardia Postural Ortostática (POTS): Uma Revisão Sistemática

**Figura Central:** Uso da Ivabradina na Terapêutica de Pacientes Portadores da Síndrome da Taquicardia Postural Ortostática (POTS): Uma Revisão Sistemática

FC: frequência cardíaca; PA: pressão arterial.

## Introdução

A Síndrome da Taquicardia Postural Ortostática (POTS, do inglês *Postural Orthostatic Tachycardia Syndrome* ) é caracterizada pela presença de sintomas de intolerância ortostática^1 , 2^ por pelo menos seis meses, associada à elevação da frequência cardíaca (FC) em 30 ou mais batimentos por minuto (bpm) em adultos e de 40 bpm ou mais em indivíduos com até 19 anos, dentro de 10 minutos após assumir a posição ativa ou inclinação da cabeça para cima.^1 - 3^ No entanto, não se observa hipotensão ortostática, ou seja, queda da pressão arterial (PA) sistólica maior ou igual a 20 mmHg e diminuição da PA diastólica superior ou igual a 10 mmHg.^2 , 3^

A prevalência da POTS ainda é desconhecida, devido ao subdiagnóstico, muitas vezes confundida com transtorno de ansiedade, depressão, estresse e síncope vasovagal.^4^ Trata-se de uma síndrome comum em mulheres brancas na idade fértil e que possui relação com outras comorbidades, como enxaqueca, síndrome do intestino irritável e síndrome de Ehlers-Danlos.^5^

São conhecidos três mecanismos fisiopatológicos que podem resultar em fenótipos distintos de POTS: neuropatia autonômica parcial, hipovolemia persistente e estado hiperadrenérgico central e que podem coexistir nos pacientes portadores de POTS.^6^ Apesar das diferenças, todos eles convergem para taquicardia e alteração da perfusão cerebral, originando as manifestações clínicas da síndrome.^7^

O principal foco no tratamento da POTS é o condicionamento físico por meio da prática de exercícios. Estudos mostram que 53% dos pacientes deixaram de preencher os critérios de POTS após três meses de prática regular de exercícios físicos.^8 , 9^ Outras intervenções não farmacológicas também são importantes, como ingestão de líquidos e sal e uso de meias de compressão e cintas abdominais.^1 , 10^ Se as abordagens não farmacológicas não forem eficazes, alguns fármacos (direcionado ao fenótipo de POTS do paciente) podem ser utilizados. Porém, até o momento, nenhum medicamento específico foi aprovado pela *Food and Drug Administration* (FDA) para o tratamento da POTS.^9^ Entre os fármacos que costumam ser utilizados estão a fludrocortisona, desmopressina, eritropoietina, midodrina, betabloqueadores, inibidores da recaptação de serotonina e norepinefrina, antagonistas combinados dos receptores alfa e beta-adrenérgicos e clonidina. Porém, os efeitos adversos desses medicamentos são um desafio para o tratamento da POTS.^10^

A ivabradina é um inibidor seletivo dos canais If ( *funny* ) de sódio presentes nas células do nó sinoatrial e age prolongando a despolarização diastólica lenta e promovendo a diminuição da FC sem afetar as demais funções cardiovasculares.^8 , 11^ Embora seja aprovada apenas para insuficiência cardíaca e angina instável, alguns estudos têm demonstrado benefício do uso da ivabradina no tratamento da síndrome de POTS. Desse modo, essa revisão sistemática buscou avaliar a eficácia e segurança do uso da ivabradina na terapêutica de pacientes portadores da POTS.

## Métodos

A revisão sistemática foi realizada seguindo-se o método *Preferred Reporting Items for Systematic Reviews and Meta-Analyses* (PRISMA). Foram buscados os descritores DeCS/MeSH “Ivabradina” e “Síndrome da Taquicardia Postural Ortostática” nas bases de dados PubMed, Scielo, LILACS e *Google Scholar.* Os critérios de exclusão foram artigos com títulos repetidos, relatos de caso, revisões sistemáticas, diretrizes e artigos originais que não estivessem em língua inglesa, espanhola ou portuguesa ou publicados há mais de 10 anos. Após a exclusão foram selecionados os artigos originais compatíveis com o tema proposto, avaliado inicialmente pelo título, depois pelo resumo e por último pela integra do artigo. Os artigos resultantes foram agrupados de acordo com o sistema PICO ( *Population, Intervention, Comparation, Outcome* ) a analisados através do questionamento sobre a eficácia e segurança do uso da ivabradina nos pacientes com POTS.

## Resultados

**Figura 1:**
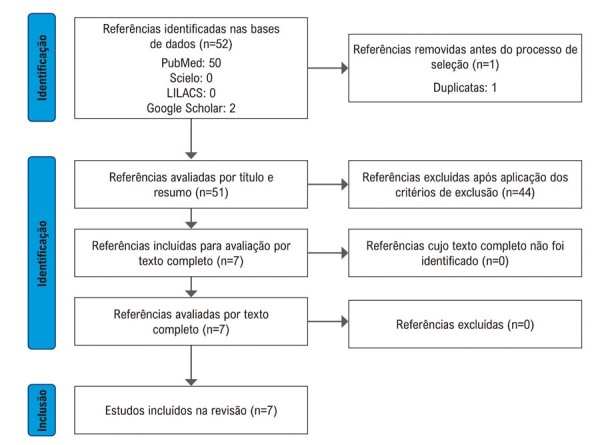
– Fluxograma PRISMA. Fonte: Elaborada pelos autores, 2024.

Foram identificados 52 artigos nas bases de dados, PubMed (50), Scielo (0), LILACS (0) e Google Scholar (2); uma duplicata foi removida, restando 51 artigos. Após aplicados os critérios de exclusão e seleção, sete artigos foram incluídos na revisão sistemática – três estudos prospectivos e quatro estudos retrospectivos ( Figura 1 ).

Entre os estudos prospectivos, havia um ensaio clínico randomizado duplo-cego controlado por placebo e dois observacionais de coorte, totalizando 85 pacientes portadores de POTS. Os retrospectivos, por sua vez, avaliaram 118 pacientes com POTS. Portanto, ao todo, foram analisados 203 pacientes ( Tabelas 1 e 2 ). Com relação aos aspectos demográficos, 153 pacientes (75,36%) eram do sexo feminino. A idade variou de 22,2 a 45,6 anos, sendo que 49 pacientes (24,1%) eram menores de 18 anos ( Figura Central )

**Tabela 1:** – Estudos prospectivos agrupados conforme a estratégia PICO

Título e autor	População	Intervenção	Comparação	Desfecho
*Randomized Trial of Ivabradine in Patients with Hyperadrenergic POTS* Taub et al.^12^	22 pacientes com POTS hiperadrenérgico. 95,5% mulheres e 86,4% brancos. Média de idade: 33,9 ± 11,7 anos	Estudo randomizado duplo-cego controlado por placebo. 1 mês de tratamento com 5 mg de placebo ou ivabradina 2 vezes por dia. Duração de 2,5 meses	Grupo ivabradina x grupo placebo. Parâmetros comparados: FC, PA, qualidade de vida e níveis de NE	Redução da FC na postura ortostática (p < 0,001), ausência de hipotensão clinicamente significativa, melhora física (p = 0,008) e social (p = 0,021) e diminuição de NE de supino para ortostático em relação à linha de base (p = 0,030)
*Ivabradine effects on COVID-19- associated POTS: a single center prospective study* Abdelnabi et al.^15^	55 pacientes com POTS associada à COVID-19. 58,2% homens. Média de idade: 30,5 ± 6,9 anos. Média de dias após o diagnóstico de COVID-19: 13,5 ± 5,6 dias.	Estudo observacional de coorte prospectivo. 7 dias de tratamento com 5 mg de ivabradina 2 vezes por dia.	Parâmetros comparados: sintomas e FC. Entre o início e o término do tratamento.	Melhora dos sintomas (palpitações) em 78% dos pacientes e da FC de 24 horas.
*The Effect of Ivabradine on the Heart Rate and Sympathovagal Balance in POTS* Barzilai and Jacob.^14^	8 pacientes com POTS. Relação 6:2 (mulheres: homens). Média de idade: 31 ± 3 anos.	Estudo prospectivo aberto sem controle de placebo. Preenchimento de questionário sobre os sintomas e aferição de FC e PA antes e após o teste com 7,5 mg de ivabradina em dose única.	Parâmetros comparados: sintomas, variação de FC e PA e da função autonômica na posição supina e em inclinação por 20 minutos. Entre o início e o término do tratamento com ivabradina.	Atenuação dos principais sintomas ortostáticos (tontura, visão turva e palpitações), redução da FC após 5 minutos de inclinação (p < 0,01). Não houve alteração da PA e do tônus vagal cardiovascular.

FC: frequência cardíaca; PA: pressão arterial; NE: norepinefrina. Obs.: Todos os estudos, adotaram 5% de significância estatística. Fonte: Elaborada pelos autores, 2024.

**Tabela 2:** – Estudos retrospectivos agrupados conforme a estratégia PICO

Título e autor	População	Intervenção	Comparação	Desfecho
*Single centre experience of ivabradine in POTS* McDonald et al.^18^	20 pacientes receberam ivabradina para tratamento de POTS de janeiro de 2008 até 2010. Relação 5:1 (mulheres:homens). Média de idade: | 35 ± 9,9 anos.	Estudo retrospectivo. Pacientes foram avaliados através de questionário (14) e/ou pela revisão do prontuário (8). Onze permaneciam em uso de ivabradina e 9 haviam descontinuado.	Parâmetros comparados: sintomas e efeitos colaterais. Entre os pacientes que permaneceram usando ivabradina e aqueles que a descontinuaram	Menos episódios de palpitações e taquicardia nos pacientes que mantiveram a ivabradina (55% dos casos). Não houve redução da PA. Seis descontinuaram pela não melhora dos sintomas. Cinco relataram efeitos colaterais.
*Ivabradine in children with POTS: a retrospective study* Towheed et al.^16^	27 pacientes menores de 18 anos que receberam ivabradina de janeiro de 2015 a fevereiro de 2019. 92,5% mulheres. Média de idade: 17 anos.	Estudo retrospectivo. Revisão de prontuários.	Parâmetros comparados: sintomas, FC e PA. Antes e depois de iniciar a ivabradina.	Em 67% melhora dos sintomas. Redução da FC entre a posição sentada e ortostática sem mudança na PA. 1 paciente teve bradicardia grave.
*Ivabradine in POTS: Preliminary Experience in Children* Donne et al.^17^	22 pacientes menores de 18 anos receberam ivabradina de fevereiro de 2008 a junho de 2014. Relação 15:7 (fem.: masc.). Média de idade: 14,5 anos.	Estudo retrospectivo. Análise de registros de FC e ECG antes do uso da ivabradina e durante o seguimento.	Parâmetros comparados: sintomas, FC e intervalo QTc. Antes de iniciar o tratamento com ivabradina e durante.	Redução da FC de repouso de 82,5 bpm para 71,3 bpm. Nenhum intervalo QTc anormal. Melhora dos sintomas em 2/3 dos pacientes.
*Ivabradine in the treatment of POTS, a single center experience* Ruzieh et al.^13^	49 pacientes que receberam ivabradina entre janeiro de 2010 e outubro de 2016. 95,9% mulheres. Média de idade: 35,1 ± 10,35 anos.	Estudo retrospectivo. Consultas e ligações telefônicas com pacientes que receberam 5 mg de ivabradina por dia para tratar POTS.	Parâmetros comparados: sintomas e efeitos adversos. Antes e depois do início do tratamento.	88,4% com melhora das palpitações. 76% com melhora dos sintomas. Ausência de efeitos adversos. Redução da FC em ortostase (p < 0,001) e sentado (p = 0,01) sem alteração da PA.

FC: frequência cardíaca; PA: pressão arterial; ECG: eletrocardiograma; Obs.: Todos os estudos, adotaram 5% de significância estatística. Fonte: Elaborada pelos autores, 2024.

Os estudos analisados demonstraram que, com o uso da ivabradina, houve redução estatisticamente significativa da FC sem alteração da PA e melhora dos sintomas de intolerância ortostática. A maioria dos pacientes não relatou efeitos adversos, embora um indivíduo tenha apresentado bradicardia grave após a administração da ivabradina.

O estudo de Taub et al.^12^ foi o único que mostrou uma redução estatisticamente significativa da FC durante o tratamento com ivabradina em comparação a placebo em pacientes com POTS hiperadrenérgico. Esse estudo também avaliou que os níveis plasmáticos de noradrenalina medidos entre as posições supina e ereta foram menores com a administração da ivabradina quando comparados ao placebo.^12^ Em outro estudo, cerca de 78% da coorte apresentaram melhora significativa dos sintomas e não tiveram efeitos adversos consideráveis. A fotopsia foi o evento mais relatado, mas nenhum dos participantes descontinuou a medicação.^13^

## Discussão

Os estudos prospectivos avaliaram a resposta clínica de pacientes com POTS que foram submetidos ao tratamento com ivabradina, comparando-a com o grupo placebo ou à condição inicial antes de iniciar o seu uso. Os estudos retrospectivos analisaram os indivíduos que já utilizavam a ivabradina para POTS ou descontinuaram seu uso por alguma razão. Na maioria desses estudos, os pacientes foram avaliados por meio questionários, prontuários, consultas, ligações telefônicas e registros de FC e eletrocardiografia. Dessa forma, os estudos prospectivos foram mais úteis na análise de eficácia e os retrospectivos na demonstração da segurança do uso da ivabradina. O pequeno número de pacientes avaliados, principalmente nos estudos prospectivos, e a maneira como os dados foram estatisticamente avaliados não permitiram a realização de uma metanálise robusta e confiável.

Na análise dos estudos isolados, o de Taub et al.^12^ foi o mais impactante, mostrando uma redução significativa da FC com a ivabradina. Porém, cabe lembrar que eram pacientes com POTS hiperadrenérgico, e que o tratamento dos pacientes deve respeitar o perfil fenotípico e fisiopatológico. Esse resultado pode ser atribuído à capacidade da ivabradina em reduzir a FC sem causar hipotensão e da possível propriedade de *down-regulation* do sistema nervoso simpático. Nesse estudo, observaram-se níveis plasmáticos normais de noradrenalina corroborando o resultado observado.^12^

Assim como no estudo de Taub et al.,^12^ os principais parâmetros analisados pelos artigos foram FC, PA, sintomas e efeitos adversos. Foram comparadas as condições antes e após o início da ivabradina, no caso dos estudos de coorte. Quanto aos estudos prospectivos, dois apresentaram redução estatisticamente significativa da FC na posição ortostática ou inclinação, e ausência de hipotensão ortostática,^12 , 14^e um relatou diminuição da FC de 24 horas.^15^ Com exceção do estudo de Taub et al.,^12^ a maioria dos estudos não especificou o principal componente da POTS. Assim, poderíamos inferir que o uso da ivabradina, beneficiaria não só os hiperadrenérgicos, como também os pacientes com outros fenótipos ou fisiopatologia.

Os estudos retrospectivos também verificaram uma redução da FC. Em dois estudos, entre a ortostase e a posição sentada, não se observou alteração da PA.^13 , 16^ Donne et al.^17^ observaram uma queda da FC de repouso de 82,5 bpm para 71,3 bpm. No geral, os pacientes que utilizaram a ivabradina apresentaram menos taquicardia, o que corrobora o uso do medicamento em seu tratamento. Além disso, a maioria dos pacientes relatou melhora dos sintomas, incluindo palpitações. Do total, seis descontinuaram a ivabradina devido à não melhora dos sintomas e seis relataram efeitos adversos, sendo a bradicardia grave em um deles. Esses fatos sustentam a segurança do uso da ivabradina na POTS.

Apesar do pequeno número de estudos e de pacientes, os resultados sobre o uso da ivabradina nos pacientes portadores de POTS parecem promissores, indicando eficácia e segurança da medicação, com melhora da taquicardia e dos sintomas de intolerância ortostática, sem comprometimento da PA e sem efeitos adversos significativos.

## Conclusão

O uso da ivabradina na terapêutica dos pacientes portadores de POTS mostrou-se eficaz e seguro. Houve diminuição da FC sem comprometimento de outras funções hemodinâmicas e alívio de sintomas relacionados à intolerância ortostática. Não se observaram efeitos adversos significativos na maioria dos casos. Os resultados são encorajadores, porém ainda são necessários novos ensaios clínicos randomizados para garantir a segurança do medicamento.
