# Supplementary material for: Uso da Ivabradina na Terapêutica de Pacientes Portadores da Síndrome da Taquicardia Postural Ortostática (POTS): Uma Revisão Sistemática
Source: Arq Bras Cardiol. 2025 Oct 8;122(11):e20250347. [Article in Portuguese] doi: 10.36660/abc.20250347 (PMC12711217; doi:10.36660/abc.20250347)
Supplement: Translation

# Translation: Use of Ivabradine in the Treatment of Patients with Postural Orthostatic Tachycardia Syndrome (POTS): A Systematic Review

**DOI:** 10.36660/abc.20250347i

**Authors:** Ana Paula Giannella de Melo, Miguel Antônio Moretti, Antonio Carlos Palandri Chagas

**Affiliations:** 1 Brazil Faculdade de Medicina do ABC, Santo André, SP – Brazil; 2 Brazil Hospital das Clínicas da Faculdade de Medicina da Universidade de São Paulo (HCFMUSP), São Paulo, SP – Brazil

**Keywords:** Ivabradine, Postural Orthostatic Tachycardia Syndrome

## Abstract

**Background:**

Postural Orthostatic Tachycardia Syndrome (POTS) is an autonomic dysfunction characterized by symptoms of orthostatic intolerance, associated with an increase in heart rate within 10 minutes of assuming an upright position or head-up tilt, in the absence of hypotension. There are three phenotypes of POTS – neuropathic, hypovolemic, and hyperadrenergic – and all result in tachycardia and altered cerebral perfusion. Pharmacological therapy is indicated in certain cases; however, no specific medication has yet been approved for this condition. Some studies have shown that ivabradine may be beneficial, as it reduces heart rate without affecting blood pressure.

**Objectives:**

To evaluate the efficacy and safety of ivabradine in the treatment of POTS.

**Method:**

Systematic review using the descriptors “Ivabradine” and “Postural Orthostatic Tachycardia Syndrome” in the PubMed, Scielo, LILACS, and Google Scholar databases. Articles were grouped and assessed using the PICO strategy.

**Results:**

A total of 52 articles were identified, of which seven were included in the review – three prospective and four retrospective studies. In total, 203 patients were evaluated, the majority of whom were female. All studies reported a significant reduction in heart rate and improvement in symptoms of orthostatic intolerance, with most patients not reporting adverse effects, regardless of their POTS phenotype.

**Conclusion:**

Ivabradine proved to be effective and safe in the treatment of patients with POTS.

## Introduction

Postural Orthostatic Tachycardia Syndrome (POTS) is characterized by the presence of symptoms of orthostatic intolerance^1 , 2^for at least six months, associated with an increase in heart rate (HR) of 30 beats per minute (bpm) or more in adults, and 40 bpm or more in individuals up to 19 years old, within 10 minutes of assuming an upright position or head-up tilt.^1 - 3^However, orthostatic hypotension is not observed – that is, a drop in systolic blood pressure (BP) of 20 mmHg or more and a decrease in diastolic BP of 10 mmHg or more.^2 , 3^

The prevalence of POTS remains unknown due to underdiagnosis, as it is often mistaken for anxiety disorders, depression, stress, or vasovagal syncope.^4^It is a syndrome commonly seen in white women of childbearing age and is associated with other comorbidities such as migraine, irritable bowel syndrome, and Ehlers-Danlos syndrome.^5^

Three pathophysiological mechanisms are known to result in distinct POTS phenotypes: partial autonomic neuropathy, persistent hypovolemia, and a central hyperadrenergic state, which may coexist in patients with POTS.^6^Despite their differences, all mechanisms converge toward tachycardia and altered cerebral perfusion, leading to the clinical manifestations of the syndrome.^7^

The primary focus in the treatment of POTS is physical conditioning through regular exercise. Studies have shown that 53% of patients no longer met the diagnostic criteria for POTS after three months of consistent physical activity.^8 , 9^Other non-pharmacological interventions are also important, such as increased fluid and salt intake, and the use of compression stockings and abdominal binders.^1 , 10^If non-pharmacological approaches prove ineffective, certain medications – tailored to the patient’s POTS phenotype – may be considered. However, to date, no specific drug has been approved by the Food and Drug Administration (FDA) for the treatment of POTS.^9^Commonly used medications include fludrocortisone, desmopressin, erythropoietin, midodrine, beta-blockers, serotonin and norepinephrine reuptake inhibitors, combined alpha and beta-adrenergic receptor antagonists, and clonidine. Nevertheless, the adverse effects of these drugs pose a challenge in the management of POTS.^10^

Ivabradine is a selective inhibitor of the If (funny) sodium channels found in sinoatrial node cells. It works by prolonging slow diastolic depolarization and reducing HR without affecting other cardiovascular functions.^8 , 11^Although currently approved only for heart failure and unstable angina, some studies have shown benefits of ivabradine in the treatment of POTS. Therefore, this systematic review aimed to evaluate the efficacy and safety of ivabradine in the management of patients with POTS.

## Methods

The systematic review was conducted following the Preferred Reporting Items for Systematic Reviews and Meta-Analyses (PRISMA) methodology. The DeCS/MeSH descriptors “Ivabradine” and “Postural Orthostatic Tachycardia Syndrome” were searched in the PubMed, Scielo, LILACS, and Google Scholar databases. Exclusion criteria included duplicate titles, case reports, systematic reviews, guidelines, and original articles not published in English, Spanish, or Portuguese, or published more than 10 years ago. After applying the exclusion criteria, original articles relevant to the proposed topic were selected, initially based on the title, followed by the abstract, and finally the full text. The resulting articles were grouped according to the PICO system (Population, Intervention, Comparison, Outcome) and analyzed to assess the efficacy and safety of Ivabradine in patients with POTS.

## Results

A total of 52 articles were identified across the databases: PubMed (50), Scielo (0), LILACS (0), and Google Scholar (2). One duplicate was removed, leaving 51 articles. After applying the exclusion and selection criteria, seven articles were included in the systematic review—three prospective studies and four retrospective studies ( Figure 1 ).

**Figure 1:** – PRISMA flowchart (prepared by the authors), 2024.

Among the prospective studies, there was one randomized double-blind placebo-controlled clinical trial and two observational cohort studies, totaling 85 patients diagnosed with POTS. The retrospective studies, in turn, evaluated 118 patients with POTS. Therefore, a total of 203 patients were analyzed ( Tables 1 and 2 ). Regarding demographic aspects, 153 patients (75.36%) were female. Age ranged from 22.2 to 45.6 years, with 49 patients (24.1%) under 18 years old ( Central Illustration ).

**Table 1:** – Prospective studies grouped according to the PICO strategy

Títle and author	Population	Intervention	Comparison	Outcome
*Randomized Trial of Ivabradine in Patients with Hyperadrenergic POTS* Taub et al.^12^	22 patients with hyperadrenergic POTS. 95.5% were female and 86.4% were White. Mean age: 33.9 ± 11.7 years	Randomized double-blind placebo-controlled study. One month of treatment with 5 mg of placebo or ivabradine twice daily. Total duration: 2.5 months	Ivabradine group vs. placebo group. Parameters compared: HR, BP, quality of life, and norepinephrine (NE) levels	Significant reduction in HR in the upright posture (p < 0.001), no clinically significant hypotension, improvement in physical functioning (p = 0.008) and social functioning (p = 0.021), and a decrease in NE from supine to upright compared to baseline (p = 0.030).
*Ivabradine effects on COVID-19- associated POTS: a single center prospective study* Abdelnabi et al.^15^	55 patients with COVID-19–associated POTS. 58.2% male. Mean age: 30.5 ± 6.9 years. Mean number of days after COVID-19 diagnosis: 13.5 ± 5.6 days.	Prospective observational cohort study. Seven days of treatment with 5 mg of ivabradine twice daily.	Parameters compared: symptoms and HR, between the beginning and end of treatment	Improvement in symptoms (palpitations) in 78% of patients and reduction in 24-hour HR
*The Effect of Ivabradine on the Heart Rate and Sympathovagal Balance in POTS* Barzilai and Jacob.^14^	8 patients with POTS. Female-to-male ratio: 6:2. Mean age: 31 ± 3 years	Open-label prospective study without placebo control. Patients completed a symptom questionnaire and had HR and BP measured before and after a single 7.5 mg dose of ivabradine	Parameters compared: symptoms, changes in HR and BP, and autonomic function in the supine position and during a 20-minute tilt	Attenuation of key orthostatic symptoms (dizziness, blurred vision, and palpitations), significant reduction in HR after 5 minutes of tilt (p < 0.01). No changes in BP or cardiovascular vagal tone were observed

HR: heart rate; BP: blood pressure; NE: norepinephrine. Note: All studies adopted a 5% level of statistical significance. Source: Prepared by the authors, 2024.

**Table 2:** – Retrospective studies grouped according to the PICO strategy

Títle and author	Population	Intervention	Comparison	Outcome
*Single centre experience of ivabradine in POTS* McDonald et al.^18^	20 patients received ivabradine for the treatment of POTS between January 2008 and 2010. Female-to-male ratio: 5:1. Mean age: 35 ± 9.9 years.	Retrospective study.Patients were evaluated through questionnaires (14) and/or medical record review (8). Eleven continued using ivabradine, while nine had discontinued.	Parameters compared: symptoms and side effects between patients who continued ivabradine and those who discontinued.	Fewer episodes of palpitations and tachycardia were reported among patients who remained on ivabradine (55% of cases). No reduction in BP was observed. Six patients discontinued due to lack of symptom improvement. Five reported side effects.
*Ivabradine in children with POTS: a retrospective study* Towheed et al.^16^	27 patients under 18 years of age received ivabradine from January 2015 to February 2019. 92.5% were female. Mean age: 17 years.	Retrospective study: chart review.	Parameters compared: symptoms, HR, and BP before and after starting ivabradine.	Symptom improvement in 67% of patients. Reduction in HR between seated and upright positions without changes in BP. One patient experienced severe bradycardia.
*Ivabradine in POTS: Preliminary Experience in Children* Donne et al.^17^	22 patients under 18 years of age received ivabradine from February 2008 to June 2014. Female-to-male ratio: 15:7. Mean age: 14.5 years.	Retrospective study. Analysis of HR and ECG records before starting ivabradine and during follow-up.	Parameters compared: symptoms, HR, and QTc interval before and during ivabradine treatment.	Resting HR decreased from 82.5 bpm to 71.3 bpm. No abnormal QTc intervals were observed. Symptom improvement was reported in two-thirds of the patients
*Ivabradine in the treatment of POTS, a single center experience* Ruzieh et al.^13^	49 patients received ivabradine between January 2010 and October 2016. 95.9% were female. Mean age: 35.1 ± 10.35 years.	Retrospective study. Consultations and phone calls with patients who received 5 mg of ivabradine daily for the treatment of POTS.	Parameters compared: symptoms and adverse effects before and after starting treatment.	88.4% reported improvement in palpitations, 76% reported overall symptom improvement. No adverse effects were observed. HR was significantly reduced in the upright position (p < 0.001) and while seated (p = 0.01), with no change in BP.

HR: heart rate; BP: blood pressure; ECG: electrocardiogram. Note: All studies adopted a 5% level of statistical significance. Source: Prepared by the authors, 2024.

The analyzed studies demonstrated that treatment with ivabradine resulted in a statistically significant reduction in HR without changes in BP, along with improvement in symptoms of orthostatic intolerance. Most patients did not report adverse effects, although one individual experienced severe bradycardia following ivabradine administration.

The study by Taub et al.^1 2^was the only one to show a statistically significant reduction in HR during treatment with ivabradine compared to placebo in patients with hyperadrenergic POTS. This study also evaluated plasma norepinephrine levels measured in supine and upright positions, which were lower with ivabradine administration compared to placebo.^1 2^In another study, approximately 78% of the cohort showed significant symptom improvement and did not experience notable adverse effects. Photopsia was the most commonly reported event, but none of the participants discontinued the medication.^1 3^

## Discussion

Prospective studies evaluated the clinical response of patients with POTS who underwent treatment with ivabradine, comparing it to a placebo group or to their baseline condition prior to starting the medication. Retrospective studies, on the other hand, analyzed individuals who were already using ivabradine for POTS or had discontinued its use for some reason. In most of these studies, patients were assessed through questionnaires, medical records, consultations, phone calls, and HR and electrocardiography recordings. Thus, prospective studies were more useful for analyzing efficacy, while retrospective studies contributed to demonstrating the safety of ivabradine use. The small number of patients evaluated, especially in prospective studies, and the way the data were statistically analyzed did not allow for a robust and reliable meta-analysis.

Among the individual studies, the one by Taub et al.^1 2^was the most impactful, showing a significant reduction in HR with ivabradine. However, it is important to note that these were patients with hyperadrenergic POTS, and treatment should be tailored to the phenotypic and pathophysiological profile of each patient. This result may be attributed to ivabradine’s ability to reduce HR without causing hypotension, as well as its possible down-regulation effect on the sympathetic nervous system. In that study, normal plasma norepinephrine levels were observed, supporting the findings.^12^

As in the study by Taub et al.,^12^the main parameters analyzed across the articles were HR, BP, symptoms, and adverse effects. In the cohort studies, conditions before and after the initiation of ivabradine were compared. Among the prospective studies, two showed a statistically significant reduction in HR in the upright or tilted position, with no orthostatic hypotension observed,^12 , 14^and one reported a decrease in 24-hour HR.^15^Except for the study by Taub et al.,^1 2^most studies did not specify the predominant POTS phenotype. Therefore, it can be inferred that ivabradine may benefit not only hyperadrenergic patients but also those with other phenotypes or pathophysiological profiles.

The retrospective studies also demonstrated a reduction in HR. In two studies, no changes in BP were observed between the upright and seated positions.^13 , 16^Donne et al.^17^reported a decrease in resting HR from 82.5 bpm to 71.3 bpm. Overall, patients treated with ivabradine experienced less tachycardia, supporting the use of the drug in POTS management. Additionally, most patients reported symptom improvement, including reduced palpitations. In total, six patients discontinued ivabradine due to lack of symptom improvement, and six reported adverse effects, with one case of severe bradycardia. These findings support the safety of ivabradine in POTS treatment.

Despite the small number of studies and patients, the results regarding ivabradine use in individuals with POTS appear promising, indicating both efficacy and safety of the medication, with improvements in tachycardia and orthostatic intolerance symptoms, without compromising BP and with no significant adverse effects.

## Conclusion

The use of ivabradine in the treatment of patients with POTS has proven to be effective and safe. There was a reduction in HR without compromising other hemodynamic functions, along with relief of symptoms related to orthostatic intolerance. No significant adverse effects were observed in most cases. The results are encouraging; however, further randomized clinical trials are still needed to ensure the safety of the medication.
